# Cleft Lip and Palate Classification Through Vision Transformers and Siamese Neural Networks

**DOI:** 10.3390/jimaging10110271

**Published:** 2024-10-25

**Authors:** Oraphan Nantha, Benjaporn Sathanarugsawait, Prasong Praneetpolgrang

**Affiliations:** School of Information Technology, Sripatum University, Bangkok 10900, Thailand; oraphan.nan@spumail.net (O.N.); benjaporn.sa@spu.ac.th (B.S.)

**Keywords:** cleft lip and palate, vision transformers, siamese neural networks, few-shot learning, medical assessment

## Abstract

This study introduces a novel approach for the diagnosis of Cleft Lip and/or Palate (CL/P) by integrating Vision Transformers (ViTs) and Siamese Neural Networks. Our study is the first to employ this integration specifically for CL/P classification, leveraging the strengths of both models to handle complex, multimodal data and few-shot learning scenarios. Unlike previous studies that rely on single-modality data or traditional machine learning models, we uniquely fuse anatomical data from ultrasound images with functional data from speech spectrograms. This multimodal approach captures both structural and acoustic features critical for accurate CL/P classification. Employing Siamese Neural Networks enables effective learning from a small number of labeled examples, enhancing the model’s generalization capabilities in medical imaging contexts where data scarcity is a significant challenge. The models were tested on the UltraSuite CLEFT dataset, which includes ultrasound video sequences and synchronized speech data, across three cleft types: Bilateral, Unilateral, and Palate-only clefts. The two-stage model demonstrated superior performance in classification accuracy (82.76%), F1-score (80.00–86.00%), precision, and recall, particularly distinguishing Bilateral and Unilateral Cleft Lip and Palate with high efficacy. This research underscores the significant potential of advanced AI techniques in medical diagnostics, offering valuable insights into their application for improving clinical outcomes in patients with CL/P.

## 1. Introduction

Cleft lip and/or palate (CL/P) is a prevalent congenital deformity that affects approximately one in every 700 to 1000 births globally [1]. This condition, characterized by its striking visual impact and potentially severe functional implications, arises from the incomplete fusion of facial structures during critical stages of fetal development. The result is a split or opening in the upper lip and/or palate, which can manifest in varying degrees of severity. The spectrum of this anomaly is remarkably wide, ranging from a minor, almost imperceptible notch in the lip to a significant, bilateral opening that creates a direct connection between the oral and nasal cavities. Such extensive clefts can dramatically alter facial symmetry and structure, significantly impairing normal facial appearance and functionality. The complexity of CL/P necessitates a comprehensive, multidisciplinary approach to treatment, typically encompassing multiple surgical interventions beginning in infancy and extending well into adolescence. These procedures are complemented by long-term rehabilitative therapies designed to address both the cosmetic and functional impairments associated with the condition. Treatment goals extend far beyond aesthetic concerns, aiming to resolve challenges with essential functions such as breathing, hearing, and speech production. Moreover, the psychological impact of CL/P on affected individuals and their families cannot be overstated, underscoring the importance of holistic care that addresses both physical and emotional well-being throughout the treatment journey.

The etiology of CL/P is a subject of ongoing research and debate within the medical community, as it is not fully understood but is widely believed to be caused by a complex interplay of genetic predispositions and environmental factors [1]. This multifactorial origin presents significant challenges in both prevention and treatment strategies. Extensive research indicates that the occurrence of CL/P is influenced by a myriad of maternal health factors and environmental conditions during crucial periods of pregnancy. These factors include, but are not limited to, nutritional deficiencies (particularly folic acid and vitamin B12), exposure to environmental toxins and teratogens, certain medications (such as anticonvulsants), and lifestyle choices including maternal smoking and alcohol consumption [1]. The impact of these environmental factors is thought to be modulated by genetic susceptibility, highlighting the intricate relationship between nature and nurture in the development of this condition. Furthermore, certain genetic syndromes and specific gene mutations have been definitively linked to an increased risk of CL/P, suggesting a strong hereditary component in some cases. Notable examples include Van der Woude syndrome and mutations in the IRF6 gene, which have been associated with both syndromic and non-syndromic forms of CL/P. This complex, multifactorial causation significantly complicates both diagnosis and treatment approaches, emphasizing the critical need for advanced diagnostic tools, including genetic screening and 3D imaging technologies, to identify at-risk pregnancies and facilitate early intervention. Moreover, the varied etiology of CL/P underscores the importance of developing personalized treatment plans that take into account the unique genetic and environmental factors contributing to each individual case, potentially paving the way for more targeted and effective therapeutic strategies in the future.

Recent advancements in medical technology, particularly in the rapidly evolving field of artificial intelligence (AI), have begun to play an increasingly crucial role in the comprehensive management of cleft lip and/or palate (CL/P). AI technologies, encompassing sophisticated approaches such as machine learning and deep learning, are being progressively integrated into various aspects of CL/P care, from initial diagnosis to treatment planning and long-term outcome assessment [2]. These innovative technologies offer the potential to revolutionize the way healthcare professionals approach CL/P, providing tools that can enhance diagnostic accuracy, optimize treatment strategies, and improve overall patient outcomes. The application of AI in this domain is multifaceted, ranging from automated image analysis for early detection of CL/P in prenatal ultrasounds to predictive modeling for surgical outcomes and personalized treatment planning. Among these cutting-edge AI technologies, Vision Transformers (ViTs) and Siamese Neural Networks have emerged as particularly promising approaches in the realm of medical imaging and diagnostics. These advanced models have demonstrated remarkable capabilities in analyzing complex visual data with high precision and efficiency, attributes that are especially valuable in the context of CL/P management where subtle anatomical details can have significant implications for treatment decisions [3,4]. The integration of these AI technologies into clinical practice represents a paradigm shift in CL/P care, offering the potential to augment human expertise with data-driven insights and improve the overall quality of patient care.

Vision Transformers (ViTs), a groundbreaking class of deep learning models inspired by the success of transformer architectures in natural language processing, have rapidly gained traction in the field of computer vision due to their exceptional performance in image analysis tasks [3]. Unlike traditional convolutional neural networks, ViTs employ mechanisms of self-attention to process images as sequences of patches, allowing them to capture long-range dependencies and complex spatial relationships within visual data [3]. This unique approach makes ViTs particularly well-suited for tasks that require detailed and nuanced image analysis, such as the evaluation of ultrasound videos of speech utterances in patients with CL/P. The ability of ViTs to discern subtle visual cues that might elude traditional imaging techniques or human observers holds immense potential for enhancing the diagnostic process in CL/P cases. By leveraging large datasets of annotated ultrasound videos, ViTs can be trained to recognize and categorize specific characteristics associated with CL/P, including the precise degree and nature of the cleft, the positioning of the soft palate, and the movement patterns of the velopharyngeal mechanism during speech [5]. This level of detailed analysis is critical for developing accurate diagnoses and tailoring interventions to the unique needs of each patient. Moreover, the application of ViTs in CL/P management extends beyond initial diagnosis; these models can potentially assist in monitoring treatment progress over time, assessing the efficacy of surgical interventions, and predicting long-term outcomes based on early imaging data. As research in this area continues to advance, the integration of ViT technology into clinical practice promises to enhance the precision and effectiveness of CL/P management, ultimately improving the quality of life for affected individuals.

Siamese Neural Networks, renowned for their remarkable efficacy in few-shot learning scenarios, represent another cutting-edge AI architecture that holds immense potential for advancing the study and management of cleft lip and/or palate (CL/P) [4]. These innovative networks are distinguished by their unique approach to learning, which involves comparing pairs of input examples to discern and learn discriminative features. This methodology makes Siamese Networks particularly well-suited for classification tasks in domains where only limited examples of each class are available, such as the nuanced categorization of different types of CL/P from relatively small datasets [4]. The ability to effectively learn from fewer examples is a crucial advantage in the context of CL/P research and clinical practice, where large, diverse datasets can be challenging to compile due to the relative rarity of certain cleft types and the ethical considerations surrounding data collection from pediatric populations. By leveraging Siamese Networks, we can potentially overcome these data limitations, enabling more robust and accurate classification of CL/P subtypes. This capability can greatly enhance the speed and precision of CL/P diagnosis and classification, facilitating more rapid and personalized treatment decisions. Moreover, the adaptive nature of Siamese Networks allows for continuous refinement of classification models as new data becomes available, ensuring that diagnostic tools remain current and effective over time. The integration of Siamese Neural Networks into CL/P management protocols could thus represent a significant leap forward in tailoring interventions to the specific needs of each patient, potentially improving both short-term treatment outcomes and long-term quality of life for individuals affected by CL/P.

This research endeavor aims to harness the immense potential of Vision Transformers and Siamese Neural Networks for the challenging task of few-shot classification of different types of cleft lip and/or palate—specifically complete, unilateral, and bilateral clefts—utilizing the CLEFT dataset derived from ultrasound videos of speech utterances [5]. By integrating these advanced AI techniques, we seek to push the boundaries of current diagnostic capabilities in CL/P management. Our primary objective extends beyond merely improving diagnostic precision; we aspire to contribute significantly to the development of more effective, personalized treatment protocols for individuals afflicted with CL/P. The potential impact of this research is multifaceted and far-reaching. Firstly, enhanced classification accuracy could lead to earlier and more precise diagnoses, potentially enabling interventions at critical developmental stages that could mitigate some of the more severe functional impairments associated with CL/P. Secondly, by tailoring treatment approaches based on the specific type and characteristics of each cleft, surgeons and therapists could optimize their interventions, potentially reducing the number of surgical procedures required and improving overall outcomes. Furthermore, the insights gained from this AI-driven analysis of ultrasound data could shed new light on the biomechanics of speech production in individuals with CL/P, informing more targeted and effective speech therapy strategies. Ultimately, the successful implementation of these advanced AI techniques in CL/P management could lead to significantly improved functional and aesthetic outcomes for patients [2,3,6]. This, in turn, has the potential to profoundly enhance the quality of life for individuals with CL/P, not only in terms of physical health and functionality but also in psychosocial aspects, including self-esteem, social integration, and overall well-being. As this research progresses, it holds the promise of ushering in a new era of precision medicine in the field of craniofacial anomalies, where AI-assisted diagnostics and personalized treatment plans become the standard of care, offering hope for better outcomes and brighter futures for those affected by CL/P.

Despite advancements, previous studies have often been limited by reliance on single-modality data, such as speech audio [6] or imaging alone [7], and have used traditional machine learning models that may not fully capture the complex interplay between anatomical and functional aspects of CL/P. Furthermore, many existing models require large datasets for training, which is challenging in medical imaging contexts where data scarcity is common due to privacy concerns and the rarity of certain conditions.

In this study, we propose a novel method that integrates Vision Transformers (ViTs) and Siamese Neural Networks to classify different CL/P types using multimodal data from the UltraSuite CLEFT dataset [5]. Our study is the first to combine these advanced AI architectures specifically for CL/P classification. The ViTs capture global contextual information from the data, while the Siamese Neural Networks enable learning similarity metrics from limited data, effectively addressing the complexities of CL/P classification in few-shot learning scenarios. We uniquely fuse anatomical data from ultrasound images with functional data from speech spectrograms, capturing both structural and acoustic information critical for accurate classification.

Our contributions are threefold:

1. Our study is the first to integrate ViTs and Siamese Neural Networks specifically for the classification of different CL/P types using multimodal data. This integration allows the model to capture both global contextual information and learn similarity metrics from limited data, addressing the complexities of CL/P classification.
2. We uniquely fuse anatomical data from ultrasound images with functional data from speech spectrograms. This multimodal approach captures both structural and acoustic features critical for accurate CL/P classification, which has not been explored in previous studies.
3. Employing Siamese Neural Networks enables effective learning from a small number of labeled examples. This approach enhances the model’s generalization capabilities in medical imaging contexts where data scarcity is a significant challenge.

## 2. Related Works

The landscape of artificial intelligence (AI) applications in diagnosing and managing Cleft Lip and/or Palate (CL/P) has been significantly enriched by a diverse array of innovative studies, each aiming to leverage cutting-edge technologies for improved clinical outcomes in this complex field. These research endeavors span a wide spectrum of AI methodologies, from deep learning and neural networks to advanced image processing techniques, all united by the common goal of enhancing the precision and efficacy of CL/P management.

One particularly noteworthy contribution to this burgeoning field was the groundbreaking study undertaken by Wang et al. in 2019 [6]. This research team developed a sophisticated deep learning model that ingeniously combined Long Short-Term Memory (LSTM) networks with Deep Recurrent Neural Networks (DRNN) to analyze and quantify hypernasality in patients with CL/P using speech audio data. The significance of this study lies not only in its novel approach to speech analysis but also in its comprehensive utilization of a large and diverse dataset comprising Mandarin vowel sounds. This extensive dataset provided a robust foundation for training and validating the AI model, ensuring its applicability across a wide range of linguistic and phonetic variations. The results of this study were remarkably promising, with the model demonstrating an impressive accuracy rate of 93.35% in identifying and characterizing hypernasality conditions. This high level of precision underscores the immense potential of machine learning models in revolutionizing the diagnostic process for speech impairments associated with CL/P. By offering a more objective and consistent method of assessing hypernasality, this AI-driven approach could potentially standardize diagnostic criteria across different clinical settings, leading to more targeted and effective speech therapy interventions. Moreover, the success of this model in analyzing Mandarin vowel sounds opens up avenues for cross-linguistic applications, potentially benefiting CL/P patients across diverse linguistic backgrounds and enhancing the global standard of care for speech disorders related to craniofacial anomalies.

Previous studies have explored various AI methodologies for diagnosing and managing CL/P. Wang et al. [6] developed a deep learning model combining Long Short-Term Memory (LSTM) networks with Deep Recurrent Neural Networks (DRNN) to analyze hypernasality in patients with CL/P. While achieving high accuracy, their approach was limited to speech audio data and specific to hypernasality detection in Mandarin-speaking patients. This single-modality focus restricts the generalizability of their model to broader CL/P classification tasks and different linguistic contexts.

In parallel with advancements in speech analysis, significant strides have been made in the domain of anatomical imaging and analysis for CL/P management. A pioneering study in this area was conducted by Zhu et al. in 2019, focusing on the development of an automatic tongue contour tracking system using an advanced Convolutional Neural Network (CNN) framework [8]. This innovative approach represented a paradigm shift in the analysis of ultrasound images for CL/P patients, aiming to extract precise tongue contours without the need for time-consuming and potentially subjective manual annotations. The methodology employed in this study was particularly sophisticated, utilizing state-of-the-art deep learning architectures such as U-net and Dense U-net, which are renowned for their efficacy in medical image segmentation tasks. The research team’s meticulous approach extended to a comparative analysis of various loss functions, including binary cross-entropy, dice loss, and focal loss, to optimize the model’s performance. This comprehensive evaluation of different neural network architectures and loss functions resulted in a system capable of achieving substantial accuracy in tongue contour tracking. The implications of this advancement are far-reaching in the context of CL/P management. By providing detailed and reliable anatomical insights into the positioning and movement of the tongue during speech, this AI-driven approach offers clinicians a powerful tool for both diagnostic and therapeutic applications. The ability to accurately track tongue contours in real-time could significantly enhance the precision of surgical planning for cleft palate repair, allowing surgeons to tailor their approaches based on individual patient anatomy. Furthermore, in the realm of speech therapy, this technology could enable more targeted interventions by providing therapists with objective data on tongue positioning and movement during various speech tasks. This level of detailed anatomical information could lead to more personalized and effective therapy protocols, potentially accelerating the rehabilitation process for CL/P patients and improving long-term speech outcomes. The success of this study not only underscores the capability of deep learning models in providing crucial anatomical insights but also paves the way for future integrations of AI in various aspects of CL/P care, from preoperative planning to postoperative rehabilitation monitoring.

Moreover, the groundbreaking research conducted by Csapó et al. in 2022 [9] brought a highly innovative perspective to the utilization of ultrasound imaging in speech therapy, particularly focusing on the complex task of mapping articulatory movements to acoustic signals. This study represents a significant leap forward in our understanding of the intricate relationship between the physical movements of speech articulators and the resulting acoustic output, a crucial area of investigation for improving speech outcomes in patients with CL/P. The researchers embarked on a comprehensive comparative study, meticulously examining the efficacy of both wedge-shaped and raw scanline ultrasound images for articulatory-to-acoustic mapping (AAM). This dual approach to image representation allowed for a nuanced exploration of how different imaging modalities might impact the performance of deep learning models in this context. At the heart of their methodology was the implementation of a sophisticated residual network, a type of deep neural network known for its ability to handle complex, high-dimensional data with remarkable efficiency. The results of this study were particularly enlightening, suggesting that deep learning models could effectively process and analyze different ultrasound image representations without incurring significant performance losses. This finding is of paramount importance in the field of speech therapy for CL/P patients, as it highlights the flexibility and robustness of neural networks in handling various forms of complex medical imaging data. The implications of this research extend far beyond mere technical considerations; it opens up new possibilities for more personalized and effective speech therapy interventions. By demonstrating the feasibility of accurate articulatory-to-acoustic mapping using diverse ultrasound image formats, this study paves the way for the development of more sophisticated biofeedback systems in speech therapy. Such systems could potentially provide real-time visual feedback to patients, allowing them to see the movements of their articulators and understand how these movements correlate with the sounds they produce. This level of immediate, visual feedback could dramatically enhance the effectiveness of speech therapy sessions, potentially accelerating progress and improving long-term outcomes for individuals with CL/P-related speech disorders.

The field of CL/P management was further enriched by the comprehensive study conducted by Al-Hammuri et al. in 2022 [10], which explored a wide array of segmentation techniques for the precise delineation of tongue edges from ultrasound images. This research represents a critical advancement in the ongoing effort to improve the accuracy and efficiency of diagnostic imaging in CL/P cases, particularly in the context of speech assessment and therapy. The study’s methodological approach was notably thorough, encompassing a comparative analysis of both traditional image processing techniques and cutting-edge deep learning algorithms. This comprehensive evaluation allowed for a nuanced understanding of the strengths and limitations of various segmentation methods when applied to the complex task of tongue edge detection in ultrasound imagery. The findings of this study were particularly illuminating, revealing a clear superiority of deep learning techniques, especially those involving Convolutional Neural Networks (CNNs) and U-net architectures, over conventional image analysis methods. The deep learning approaches demonstrated not only superior accuracy in tongue edge delineation but also remarkable operational efficiency, potentially enabling real-time analysis in clinical settings. This marked improvement in both precision and speed has profound implications for clinical practice in CL/P management. The enhanced accuracy of tongue edge detection could lead to more precise diagnoses of speech-related issues in CL/P patients, allowing for earlier and more targeted interventions. Furthermore, the improved efficiency of these AI-driven methods could streamline clinical workflows, potentially reducing wait times for diagnoses and enabling more frequent assessments to track progress over time. The success of CNNs and U-nets in this application underscores a broader trend towards more AI-driven methodologies in clinical diagnostics, not just in CL/P management but across various medical fields. This shift towards AI-enhanced imaging analysis holds the promise of more objective, consistent, and detailed diagnostic information, which could significantly improve treatment planning and outcomes for patients with CL/P. Moreover, the ability of these deep learning models to learn and improve with exposure to more data suggests that their performance could continue to enhance over time, potentially leading to ever more accurate and reliable diagnostic tools in the future.

The imperative for the current study emerges from foundational works that have significantly contributed to the field of cleft lip and palate (CL/P) management but have also revealed opportunities for further advancement. Maier et al. [7] pioneered automatic speech assessment for children with CL/P using acoustic features and machine learning algorithms. Their system, while groundbreaking in aligning closely with expert evaluations, did not integrate imaging techniques, which limits the ability to fully assess the anatomical factors contributing to speech disorders. Millard and Richman [11] provided insights into the psychological and speech outcomes associated with different types of cleft conditions, emphasizing the need to consider the complex interplay between speech quality and psychosocial variables. However, their work relied on subjective measures rather than objective, automated diagnostic tools. Harding and Grunwell [12] presented a comprehensive review of cleft palate speech characteristics, drawing attention to the articulatory errors and developmental speech processes that arise from anatomical constraints. Although their analysis of speech patterns was thorough, it lacked the integration of advanced technologies, such as artificial intelligence (AI), to improve diagnostic accuracy. Lastly, Arasteh et al. [13] explored the impact of speech anonymization on the utility of pathological speech data, highlighting privacy concerns in clinical speech assessments. Their findings underscore the need for specialized approaches to balance privacy with the diagnostic utility of speech data, an issue that remains underexplored in the context of CL/P.

Our current study builds upon these foundational efforts by integrating Vision Transformers (ViTs) and Siamese Neural Networks to address specific challenges in CL/P management. These advanced AI architectures allow for a more nuanced analysis of both speech and anatomical data, improving diagnostic accuracy and operational efficiency while maintaining patient privacy. By combining cutting-edge imaging analysis with AI-driven speech assessment, we aim to bridge the gaps identified in previous research, ultimately enhancing clinical outcomes for CL/P patients.

## 3. Materials

### 3.1. Dataset

This section provides a detailed and comprehensive analysis of participant demographics within the Cleft Dataset, focusing on the distribution of ages and gender across different types of cleft conditions. Examining these demographic factors is crucial for understanding the representativeness of the dataset and its potential implications for research findings. Age and gender distributions can significantly influence speech production patterns, particularly in the context of developmental conditions such as cleft lip and palate. Therefore, a thorough understanding of these demographic characteristics is essential for interpreting the results of any analyses conducted on this dataset and for considering potential confounding factors in research designs. Before delving into the specifics of the dataset, it is important to understand the source of this data, the UltraSuite dataset, which provides the broader context and framework within which the Cleft Dataset is situated.

The UltraSuite dataset represents a significant advancement in speech research resources, offering a comprehensive collection of labeled ultrasound tongue imaging and synchronized audio recordings. This dataset has been meticulously curated and is primarily used for cutting-edge research in speech therapy and speech sciences [5]. The diversity and depth of UltraSuite make it an invaluable resource for researchers across various disciplines, including linguistics, speech pathology, and machine learning applied to speech analysis. It consists of several sub-datasets, each designed to cater to different research needs and objectives within the field of speech science. These include datasets focused on children with typical speech development, providing a baseline for comparative studies, as well as specialized collections for children with various speech sound disorders. This multifaceted approach allows researchers to conduct comparative analyses and develop more targeted interventions. Among these specialized collections, the Cleft Dataset stands out as a unique and particularly valuable resource for researchers focusing on the speech characteristics associated with cleft lip and palate conditions. The inclusion of both typical and atypical speech patterns within the broader UltraSuite collection enables researchers to conduct comprehensive studies that can lead to more nuanced understandings of speech production mechanisms and more effective therapeutic strategies.

The Cleft Dataset, a distinctive and specialized collection within the broader UltraSuite framework, is of particular interest and relevance to the current study. This dataset is unique in its focus on children with cleft lip and palate, providing a rich source of data for researchers investigating the specific speech production challenges associated with these congenital conditions. The dataset includes high-quality ultrasound and audio recordings that capture the intricate movements of the tongue and other articulators during speech production in children affected by various types of clefts. This level of detail is crucial for understanding the physiological basis of speech difficulties in this population and for developing targeted therapeutic interventions. The Cleft Dataset enables detailed investigations into how these congenital conditions affect various aspects of speech production, including articulation, resonance, and overall intelligibility. By providing such comprehensive data, this dataset opens up new avenues for research that can lead to significant advancements in understanding cleft-related speech disorders and in the development of more effective, evidence-based therapeutic interventions. The potential applications of this dataset extend beyond purely academic research, offering valuable insights that can directly inform clinical practice in speech therapy and contribute to improved outcomes for children with cleft lip and palate.

The dataset analyzed in this study includes data from a total of 29 children, each diagnosed with one of three specific types of cleft conditions: Bilateral Cleft Lip and Palate (BCLP), Cleft Palate only (CP), and Unilateral Cleft Lip and Palate (UCLP). This categorization is crucial as each type of cleft presents unique challenges in terms of speech production and may require different therapeutic approaches. The demographics of participants by cleft type are meticulously summarized in Table 1, which presents a comprehensive overview of the mean age, age range, standard deviation of age, along with the count of males and females for each group. This detailed breakdown allows for a nuanced understanding of the dataset’s composition and facilitates more accurate interpretations of research findings.

This detailed demographic breakdown provides several key insights into the composition of the dataset. Firstly, it reveals a relatively balanced distribution across the three cleft types, with CP and UCLP having equal representation (11 participants each) and BCLP having a slightly smaller group (7 participants). This distribution allows for meaningful comparisons between cleft types, although the smaller sample size for BCLP should be considered when interpreting results. The age ranges across all three groups are quite similar, spanning from early childhood (around 3.5 years) to pre-adolescence (around 10.5 years). This wide age range is particularly valuable as it captures a critical period in speech development and allows for potential analyses of age-related trends in speech production among children with clefts. The mean ages across groups are also relatively close, ranging from 6.04 years for CP to 7.28 years for UCLP, which facilitates more direct comparisons between groups. However, the standard deviations indicate considerable variability within each group, highlighting the importance of considering individual differences in any analyses. In terms of gender distribution, there is a notable skew towards male participants, particularly in the UCLP group. This gender imbalance should be taken into account when interpreting results and may warrant further investigation into potential gender-related differences in cleft-associated speech characteristics.

Furthermore, the Cleft Dataset is not limited to audio and ultrasound recordings but also encompasses a rich collection of textual content used during the recording sessions. This textual component of the dataset is meticulously designed and includes a diverse array of phrases and words, each carefully selected to elicit specific articulatory movements that are essential for studying speech production in individuals affected by cleft lip and palate. The inclusion of this textual data adds significant value to the dataset, as it allows researchers to correlate acoustic and articulatory data with specific linguistic contexts. This multimodal approach to data collection enables more comprehensive analyses of speech production mechanisms in children with clefts. For instance, researchers can examine how different phonetic contexts influence articulatory patterns and acoustic outputs, providing insights into the specific challenges faced by children with different types of clefts. The textual content likely includes a range of phonetic targets, such as high and low vowels, consonants requiring various places and manners of articulation, and potentially challenging sound combinations that are known to be problematic for individuals with cleft palate. This careful selection of linguistic material ensures that the dataset captures a wide spectrum of speech production phenomena relevant to cleft-related speech disorders.

The UltraSuite dataset, of which the Cleft Dataset is a crucial component, serves as a comprehensive and invaluable resource for researchers interested in exploring various aspects of speech development, speech disorders, and the effectiveness of speech therapy interventions. Its multifaceted nature allows for investigations that span from basic research in speech production to applied studies in clinical intervention strategies. The dataset provides a structured and rich set of data that includes high-quality speech waveforms, which capture the acoustic properties of speech with great precision. These waveforms are synchronized with raw ultrasound images of the tongue and oral cavity during speech production, offering unprecedented insights into the articulatory dynamics associated with different cleft types. Additionally, the dataset includes detailed ultrasound parameters, ensuring that researchers have access to all relevant technical information for accurate analysis and interpretation of the imaging data. A particularly valuable aspect of the UltraSuite dataset is the inclusion of annotated textual descriptions provided by experienced speech-language therapists. These annotations offer expert insights into the speech characteristics observed in each recording, potentially highlighting subtle features that might be overlooked in automated analyses. The combination of these diverse data types—acoustic, articulatory, and descriptive—makes UltraSuite, and particularly the Cleft Dataset, an invaluable tool for advancing research in computational linguistics, speech therapy practices, and clinical interventions related to cleft lip and palate conditions [5]. The potential applications of this dataset are vast, ranging from the development of automated diagnostic tools and personalized therapy plans to fundamental research into the neuromotor control of speech in the presence of structural abnormalities of the oral cavity.

It is important to note that the dataset exhibited a potential gender imbalance, particularly in the UCLP group where male participants were predominant. To mitigate the impact of this imbalance on the generalizability of the results, stratified *K*-fold cross-validation was employed during model training and evaluation. This approach ensured that each fold maintained consistent gender proportions reflective of the overall dataset, thereby reducing the potential bias introduced by the imbalance. Despite these measures, the gender imbalance remains a limitation of the study. The findings may not fully generalize to a more balanced population. It is recommended that future research endeavors include a more balanced dataset to enhance the robustness and applicability of the results.

### 3.2. Textual Prompts in the Cleft Dataset

The textual prompts used in the Cleft Dataset represent a carefully curated collection of words, phrases, and sentences specifically designed to target and evaluate a wide range of phonetic and articulatory features that are particularly relevant to cleft speech research. These prompts are not arbitrary; rather, they are the result of meticulous selection by speech and language experts who understand the unique challenges faced by individuals with cleft lip and palate. The design of these prompts takes into account various factors such as the typical articulatory difficulties associated with different types of clefts, the developmental stages of speech acquisition, and the specific research questions that the dataset aims to address. For instance, prompts like “asha” and “apa” are strategically crafted to evaluate the articulation of both consonants and vowels in varying phonetic environments and contexts. These simple, yet informative, syllable combinations allow researchers to isolate and analyze specific aspects of speech production, such as the ability to maintain appropriate oral-nasal resonance balance or the precision of tongue placement during articulation.

The dataset also incorporates longer, more complex phrases like “Funny Shaun is washing a dirty dish” to analyze more intricate speech patterns and natural speech flow. Such sentences enable the assessment of connected speech, providing insights into aspects like coarticulation, prosody, and overall speech intelligibility in more naturalistic contexts. These longer prompts are particularly valuable as they mimic real-world speech situations, offering a window into how children with clefts manage the complexities of continuous speech production.

The notable variability in the number of files for each prompt within the dataset is not a random occurrence but rather indicates a deliberate and focused attention on particular sounds or words that may present more significant challenges or require more detailed analysis for children with different types of cleft conditions. This structured and targeted approach serves multiple important purposes in cleft speech research. Firstly, it helps in identifying specific articulatory deficits that may be characteristic of different cleft types. For example, certain prompts may be repeated more frequently to capture the nuances of velopharyngeal function, which is often impaired in children with cleft palate. Secondly, this approach facilitates the monitoring of improvements post-therapy or surgical interventions. By having multiple recordings of the same prompts over time, researchers and clinicians can track changes in articulation accuracy, resonance quality, and overall speech intelligibility. This longitudinal perspective is invaluable for assessing the effectiveness of various treatment strategies and for tailoring interventions to individual needs. Additionally, the variation in prompt frequency allows for a balanced analysis that considers both the most challenging aspects of speech production for this population and the full range of phonetic contexts necessary for comprehensive speech assessment.

Table 2 provides a comprehensive and detailed overview of the unique textual prompts used in the Cleft Dataset recordings, including the precise number of files in which each prompt is featured. These prompts are not merely data points but are crucial tools for eliciting specific articulatory responses in children with cleft lip and palate, enabling researchers to conduct in-depth analyses of speech production mechanisms in this population.

This table not only lists the prompts but also provides valuable insights into the structure and priorities of the Cleft Dataset. The variation in the number of files for each prompt suggests a thoughtful design that balances the need for repetition of critical speech sounds with the inclusion of a diverse range of phonetic contexts. For instance, the high frequency of simple vowel-consonant-vowel (VCV) combinations like “asa” and “asha” (29 files each) indicates a strong focus on assessing fundamental articulation skills and specific phonemes that are often challenging for individuals with cleft palate. The inclusion of longer sentences and phrases, though less frequent, ensures that the dataset captures the complexities of connected speech. This comprehensive approach allows researchers to analyze both isolated speech sounds and more natural, continuous speech patterns, providing a holistic view of speech production in children with cleft lip and palate. The range of prompts also reflects consideration for different aspects of speech, including consonant production, vowel quality, nasal resonance, and prosody, making this dataset an invaluable resource for multifaceted research in cleft speech.

### 3.3. BiomedCLIP

BiomedCLIP represents a significant and transformative advancement in the domain of biomedical image processing, particularly through its innovative application in extracting and analyzing features from complex medical imaging modalities such as ultrasound. This sophisticated model is a specialized extension of the groundbreaking CLIP (Contrastive Language-Image Pre-training) architecture, meticulously tailored for the unique challenges and requirements of the biomedical domain. By leveraging large-scale, domain-specific datasets, BiomedCLIP has been designed to train robust and highly accurate vision-language models that can interpret and analyze biomedical images with unprecedented precision and depth. The development of BiomedCLIP addresses a critical need in medical image analysis, where the complexity and specificity of the data often render general-purpose computer vision models inadequate. This tailored approach ensures that the model is uniquely equipped to handle the nuances and intricacies of medical imaging, potentially revolutionizing diagnostic processes and research methodologies in fields such as radiology, pathology, and, pertinent to this study, speech and language pathology in the context of cleft lip and palate.

The pretraining process of BiomedCLIP is a cornerstone of its exceptional performance and adaptability. The model is pretrained using the extensive PMC-15M dataset, a massive collection comprising 15 million carefully curated image-text pairs derived from peer-reviewed scientific publications across various biomedical disciplines. This comprehensive and diverse training dataset is crucial in enabling BiomedCLIP to develop a deep understanding of the relationship between visual medical data and corresponding textual descriptions or annotations. The sheer scale and quality of this pretraining data allow the model to achieve superior performance in a wide array of tasks, including, but not limited to, feature extraction from complex biomedical images such as ultrasounds, MRIs, and CT scans. The extensive exposure to varied medical imaging data during pretraining equips BiomedCLIP with the ability to recognize and interpret subtle patterns and anomalies that might be overlooked by less specialized models or even human experts. This robust pretraining foundation makes BiomedCLIP particularly well-suited for transfer learning applications in specialized medical domains, such as the analysis of ultrasound images in cleft lip and palate research.

The architecture of BiomedCLIP incorporates several domain-specific adaptations that make it exceptionally well-suited for processing and interpreting medical imaging data. These adaptations are designed to address the unique challenges posed by biomedical images, such as the need for high precision, the ability to handle varied image qualities and resolutions, and the capacity to interpret complex anatomical structures. The model’s architecture includes specialized attention mechanisms and layer configurations optimized for medical image analysis. These architectural refinements enable BiomedCLIP to effectively capture and process the intricate details and subtle variations present in biomedical images, which are often critical for accurate diagnosis and research applications. The domain-specific nature of BiomedCLIP’s architecture ensures that it can extract relevant features from medical images with a level of accuracy and reliability that surpasses general-purpose computer vision models.

A key feature of BiomedCLIP is its adaptability to the typically high resolution and varied dimensions of biomedical images, which are generally larger and contain significantly more detailed information than general-domain images. To accommodate this, the input image size for BiomedCLIP is substantially adjusted and optimized. Specifically, the model is designed to process images at a much higher resolution than standard computer vision models, enabling it to capture and analyze the intricate details necessary for accurate medical analysis. This capability is particularly crucial in the context of ultrasound imaging, where subtle variations in tissue texture and structure can have significant diagnostic implications. The standard configuration of BiomedCLIP uses an image input size that is significantly larger than the typical sizes used in non-specialized vision models, often processing images at resolutions that are several times higher than those used in general computer vision tasks. This enhanced input capacity allows BiomedCLIP to maintain and analyze fine-grained details that might be lost or overlooked in models with lower input resolutions.

The feature extraction capabilities of BiomedCLIP are substantially enhanced by its utilization of a larger and more sophisticated Vision Transformer (ViT) architecture. This advanced setup includes larger patch sizes and a deeper network structure, specifically designed to process the complex and high-dimensional visual data presented by biomedical images. The model employs a patch size of 16×16 pixels, which strikes an optimal balance between capturing local details and maintaining computational efficiency. This patch size allows the model to effectively segment and analyze biomedical images, preserving important spatial relationships while enabling the processing of high-resolution inputs. Furthermore, BiomedCLIP adapts its transformer layers to effectively learn from the high-dimensional data typical of medical imaging. This adaptation involves modifications to the attention mechanisms, feed-forward networks, and normalization layers within the transformer architecture, optimizing them for the specific characteristics of biomedical image data. The deeper network structure of BiomedCLIP allows for a more nuanced and hierarchical understanding of image features, crucial for interpreting the complex anatomical structures and subtle abnormalities often present in medical images.

The final layer of BiomedCLIP is engineered to output high-dimensional feature vectors that provide a comprehensive and nuanced representation of the visual characteristics of the input images. These feature vectors are of paramount importance for subsequent tasks such as classification, detection, and detailed analysis of medical conditions visible in ultrasound images and other biomedical imaging modalities. The dimensionality of the output feature vectors is carefully designed to capture a wide array of information, ensuring that even the most subtle nuances in the images are not lost in the encoding process. This high-dimensional output is particularly crucial in medical diagnostics, where minor details can have significant clinical implications. The rich feature representations produced by BiomedCLIP enable downstream tasks to leverage a wealth of information, potentially uncovering patterns and correlations that might not be immediately apparent to human observers. This capability makes BiomedCLIP an invaluable tool in medical research and clinical practice, offering the potential to enhance diagnostic accuracy, facilitate early detection of conditions, and support more personalized treatment planning. In the context of cleft lip and palate research, these high-dimensional feature vectors could provide unprecedented insights into the subtle anatomical variations and functional characteristics associated with different types of clefts, potentially leading to more refined diagnostic criteria and targeted therapeutic interventions.

### 3.4. Few-Shot Learning with Siamese Neural Networks

Few-shot learning represents a cutting-edge machine learning paradigm specifically designed to adapt models to new tasks or classes with an extremely limited amount of labeled training data. This approach is particularly relevant and crucial in medical applications, including the diagnosis and classification of cleft lip and palate (CL/P), where obtaining large, comprehensively annotated datasets can be extraordinarily resource-intensive, ethically challenging, and often impractical due to the rarity of certain conditions or the sensitivity of medical data. The ability to learn from a small number of examples is not just a convenience in medical image analysis; it is often a necessity, given the scarcity of well-documented cases for specific pathologies or subtypes of conditions. Few-shot learning addresses this challenge by enabling models to generalize effectively from a limited number of labeled examples, potentially revolutionizing how AI systems are applied in specialized medical domains. In the context of CL/P research and diagnosis, few-shot learning offers the promise of developing highly accurate classification models even when faced with the inherent limitations of dataset size and diversity that are common in rare congenital conditions.

Siamese Neural Networks have emerged as a particularly powerful and well-suited architecture for implementing few-shot learning, especially in domains like medical imaging where distinguishing between subtle variations is crucial. The unique structure and learning approach of Siamese Networks make them exceptionally adept at learning to distinguish between classes or categories from very few examples. This capability is of paramount importance in the context of CL/P classification, where the ability to accurately differentiate between various types and severities of clefts based on a limited number of training examples can significantly enhance diagnostic accuracy and efficiency. The fundamental principle behind Siamese Networks aligns closely with the cognitive process of comparative analysis often employed by medical professionals, where new cases are evaluated by comparing them to known examples or prototypes of different conditions.

The architecture of Siamese Neural Networks is characterized by two or more identical subnetworks, a design choice that has profound implications for their learning capabilities and efficiency. These subnetworks are truly identical in every aspect, sharing the same configuration, parameters, and weights throughout the training process. This architectural symmetry is not merely a design choice but a fundamental aspect that enables Siamese Networks to learn generalizable features efficiently. During the training process, different instances or examples are fed as inputs to each subnetwork. The network then learns to differentiate between these instances, determining whether they belong to the same category or different ones. This learning process is fundamentally different from traditional neural networks, as it focuses on learning the similarity or dissimilarity between pairs of inputs rather than on classifying individual inputs into predefined categories. The shared weights across subnetworks ensure that similar inputs produce similar feature representations, regardless of which subnetwork processes them, leading to a more robust and generalizable feature space.

The learning process in Siamese Neural Networks is primarily driven by a specialized loss function known as contrastive loss. This loss function is critical in shaping the network’s ability to distinguish between paired examples effectively. The contrastive loss function is designed to penalize the network based on its performance in differentiating between pairs of inputs. It encourages the network to minimize the distance between the feature representations of inputs from the same class while maximizing the distance between representations of inputs from different classes. This approach effectively teaches the network to create a feature space where examples of the same class cluster together, and examples of different classes are pushed apart.

The general form of the contrastive loss for a Siamese Neural Network can be mathematically expressed as follows:

(1) $$L\left(\theta \right)=\sum_{\left(i,j\right)}y_{ij}D{\left(f_{\theta }\left(x_{i}\right),f_{\theta }\left(x_{j}\right)\right)}^{2}+\left(1-y_{ij}\right)\max{\left(0,m-D\left(f_{\theta }\left(x_{i}\right),f_{\theta }\left(x_{j}\right)\right)\right)}^{2}$$

where

- θ represents the parameters of the neural networks.
- $x_{i},x_{j}$ are pairs of input samples.
- $y_{ij}$ is a binary label that is 1 if $x_{i}$ and $x_{j}$ are from the same class, and 0 otherwise.
- *D* is a distance metric, typically the Euclidean distance, which measures the similarity between the outputs of the neural networks for the inputs $x_{i}$ and $x_{j}$.
- *m* is a margin that defines how far the outputs of pairs from different classes should be pushed apart.

The primary goal of the Siamese Neural Network in the context of CL/P classification is to learn a highly discriminative feature space. In this learned space, the distances between pairs of examples from the same class of cleft are minimized, while distances between pairs from different classes are maximized beyond the specified margin *m*. This approach enables the network to effectively generalize from a small amount of training data, making it exceptionally well-suited for diagnosing and classifying medical images where each instance of pathology may be unique or rare. The learned feature space becomes a powerful tool for classification, as new, unseen examples can be classified based on their proximity to known examples in this space.

By employing Siamese Neural Networks, this study leverages the power of few-shot learning to significantly enhance the accuracy and efficiency of classifying various types of cleft lip and palate. This approach demonstrates the immense potential of advanced AI methods to impact clinical diagnostics profoundly. In the context of CL/P, where subtle differences in anatomical structure can have significant implications for treatment and prognosis, the ability of Siamese Networks to learn fine-grained distinctions from limited data is particularly valuable. This methodology not only promises to improve the accuracy of CL/P classification but also opens up possibilities for more personalized treatment planning based on nuanced understanding of individual cases. Furthermore, the success of this approach in CL/P classification could pave the way for similar applications in other areas of medical imaging where rare conditions or limited data availability pose significant challenges to traditional machine learning approaches.

## 4. Methods

This section provides a comprehensive outline of the methodology employed to achieve the objectives set forth for this study. The primary aim is to leverage advanced machine learning techniques for the accurate classification of cleft lip and palate (CL/P) types using ultrasound videos and corresponding speech data. The methodology encompasses the utilization of cutting-edge Vision Transformers and Siamese Neural Networks for few-shot classification, addressing the challenges associated with multimodal data including ultrasound image sequences and speech spectrograms [3]. This approach is designed to maximize the information extracted from both visual and auditory modalities, enabling a more robust and accurate classification system. The methodology is structured into several key components: data preparation, feature extraction, model architecture, classification strategy, and evaluation metrics. Each of these components plays a crucial role in the overall framework and has been carefully designed to address the specific challenges posed by the nature of CL/P classification using multimodal data.

### 4.1. Data Preparation

Given the unique and complex nature of the data, which consists of ultrasound video sequences and audio recordings of speech, the initial step in the methodology involves extensive preprocessing of these data types to facilitate effective learning and classification [14]. This preprocessing stage is critical as it transforms the raw, unstructured data into a format that is suitable for input into advanced machine learning models. For the ultrasound video data, each sequence is meticulously sliced into *K* chunks, where the value of *K* is determined based on extensive experimentation to identify the optimal time window. This optimal window is crucial as it needs to capture relevant articulatory movements without losing important temporal context. The choice of *K* represents a balance between preserving detailed motion information and maintaining computational efficiency.

For the audio data, a sophisticated approach of generating spectrograms is employed, as they provide a comprehensive and visually interpretable representation of the speech frequency spectrum over time [15]. The spectrogram generation process can be mathematically represented as:

(2) $$\mathrm{Spectrogram}\left(S\right)={\left|\mathrm{STFT}\left(s\left(t\right)\right)\right|}^{2}$$

where STFT denotes the Short-Time Fourier Transform, and s(t) represents the original speech signal in the time domain. The resulting spectrograms are then treated as image inputs, aligning them with the modality of the ultrasound images. This alignment is crucial as it allows for a unified approach in the subsequent stages of the methodology, particularly in feature extraction and classification. The process of slicing video data and generating spectrograms is of paramount importance as it transforms the continuous video and audio streams into a structured format that is not only amenable to feature extraction but also optimized for the subsequent classification tasks. This careful preparation of data sets the foundation for the entire classification pipeline, ensuring that the maximum amount of relevant information is preserved and presented in a format that advanced machine learning models can effectively process and learn from.

### 4.2. Feature Extraction

Feature extraction is a critical component of the methodology, serving as the bridge between raw data and the high-level representations required for effective classification. For this crucial task, the state-of-the-art BiomedCLIP model configuration, which is readily available on the Hugging Face platform, is leveraged [16]. This advanced model has been specifically designed and optimized for extracting robust and meaningful features from biomedical images. Importantly, BiomedCLIP is utilized in a zero-shot setting, employing it as a feature extractor without any fine-tuning or modification of its pretrained weights.

The feature extraction process is applied systematically to each chunk of the ultrasound video and its temporally aligned spectrogram. This paired processing approach ensures that the intricate relationships between the visual articulatory movements and their acoustic manifestations are captured. The BiomedCLIP model processes this multimodal input data to generate high-dimensional feature vectors, which encapsulate the essential characteristics and latent patterns of both the visual and acoustic information.

The feature extraction process can be formally represented as:

(3) $${\mathrm{Features}}_{\mathrm{ultrasound}}^{i},{\mathrm{Features}}_{\mathrm{spectrogram}}^{i}=\mathrm{BiomedCLIP}\left({\mathrm{Image}}_{i},{\mathrm{Spectrogram}}_{i}\right)$$

In this equation, *i* serves as an index for the chunks into which the video and audio data have been divided during the preprocessing stage. Since BiomedCLIP is used in a zero-shot manner, its pretrained capability is relied upon to provide meaningful embeddings without additional training.

The resulting feature vectors are high-dimensional representations that capture the most salient aspects of the input data. These features form the foundation upon which the subsequent classification model will operate. The dimensionality and richness of these features are key to enabling the model to distinguish between the subtle variations in speech production associated with different CL/P types. By employing the BiomedCLIP model for feature extraction, the methodology benefits from the latest advancements in biomedical image processing and natural language processing. This approach allows for the extraction of features that are not only relevant to the specific task of CL/P classification but also robust to variations in imaging conditions and individual patient characteristics. The combination of visual and acoustic features provides a comprehensive representation of each patient’s speech production mechanics, setting the stage for highly accurate and reliable classification in the subsequent stages of the methodology.

### 4.3. Model Architecture

The core of the model architecture represents a sophisticated fusion of cutting-edge deep learning techniques, specifically designed to address the unique challenges posed by the multimodal, few-shot learning scenario in cleft lip and palate (CL/P) classification. At its heart, the architecture combines a Vision Transformer (ViT) for processing the image data with a Siamese Network structure, creating a powerful synergy that effectively handles the few-shot learning scenario [3,4].

The Vision Transformer component of the architecture builds upon the groundbreaking work in applying transformer models, originally designed for natural language processing, to computer vision tasks. This approach allows the model to capture long-range dependencies and global context within the ultrasound images and spectrograms, which is crucial for understanding the complex articulatory patterns associated with different CL/P types. The ViT processes the input as a sequence of patches, applying self-attention mechanisms to learn the relationships between different regions of the image or spectrogram.

The Siamese Network structure, on the other hand, is instrumental in addressing the few-shot learning aspect of the task. This architecture employs a twin network design, where two identical subnetworks with shared weights process pairs of inputs. The fundamental principle behind this approach is to learn a similarity metric between pairs of examples, rather than trying to classify them directly. This is particularly advantageous in this scenario, where there may be limited examples of each CL/P type but a need to generalize well to new, unseen cases.

In the implementation, each branch of the Siamese Network consists of a Vision Transformer that has been carefully tailored to process either the ultrasound images or the spectrograms. Crucially, the weights are shared between these branches, ensuring that both modalities (visual and acoustic) learn a compatible feature space representation. This weight sharing is a key aspect of the design, as it forces the network to find a common embedding space that captures the essential characteristics of both modalities, facilitating more robust and generalizable classification.

The learning process in the Siamese Network can be formally represented by the following loss function:

(4) $$L\left(\theta \right)=\sum_{\left(x_{1},x_{2},y\right)}\mathrm{ContrastiveLoss}\left(f_{\theta }\left(x_{1}\right),f_{\theta }\left(x_{2}\right),y\right)$$

where $x_{1},x_{2}$ are input pairs, *y* indicates if the pair is from the same class, and θ represents the network parameters.

The ContrastiveLoss function is designed to minimize the distance between the embeddings of samples from the same class while maximizing the distance between samples from different classes. This approach enables the network to learn a discriminative feature space that can effectively separate different CL/P types, even with limited training examples.

**Figure 1:**
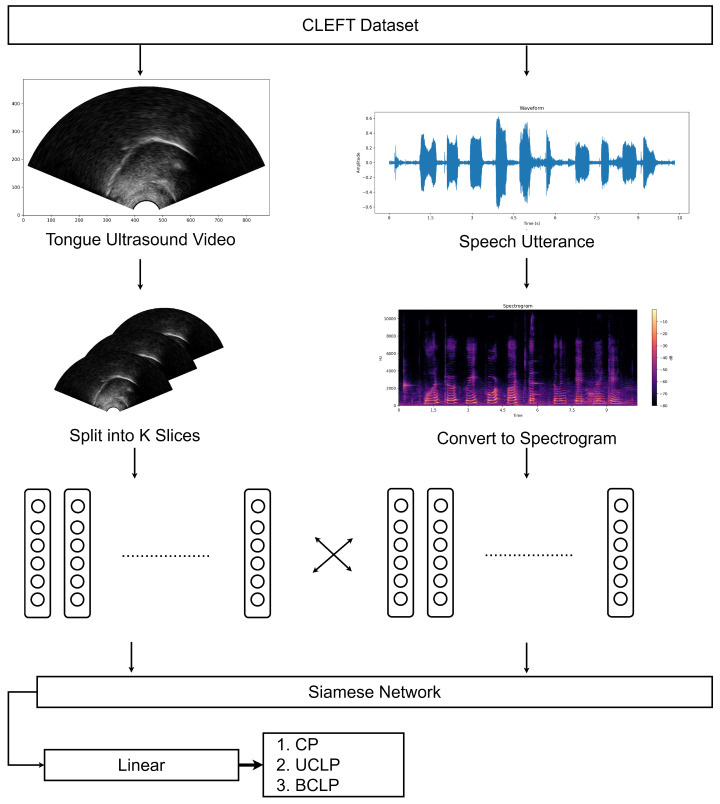
Visualization of the CLEFT Dataset used in the study, showcasing ultrasound images, waveform, spectrogram, and neural network architecture. This figure illustrates the multimodal nature of the data and the complexity of the model architecture, highlighting the integration of ultrasound imagery, acoustic waveforms, and spectrograms in the classification pipeline.

To provide a visual understanding of the methodology and the data being used, Figure 1 offers a comprehensive visualization of the CLEFT Dataset used in the study.

This figure not only demonstrates the diverse data types being worked with but also provides insight into how these different modalities are integrated into the neural network architecture. It showcases the ultrasound images, which capture the articulatory movements, alongside the corresponding speech waveforms and spectrograms, which represent the acoustic properties of the speech. The neural network architecture depicted in the figure illustrates how these diverse inputs are processed and combined to achieve the classification goals.

By leveraging this sophisticated model architecture, the aim is to capture the subtle and complex patterns that distinguish different types of CL/P, enabling accurate classification even in scenarios where training data may be limited. This approach not only addresses the challenges inherent in this specific task but also contributes to the broader field of multimodal, few-shot learning in medical image analysis.

The choice of a 16×16 patch size in the Vision Transformer is consistent with the configuration of the BiomedCLIP model [16], ensuring compatibility and effective utilization of pretrained weights. This patch size is optimal for capturing fine-grained spatial details in ultrasound images and spectrograms. In the context of cleft classification, capturing these details is critical, as subtle anatomical variations in the orofacial structures can significantly impact the classification accuracy. By using smaller patches, the model can learn more detailed representations of the input images, leading to improved performance.

#### 4.3.1. Why Vision Transformers?

Vision Transformers (ViTs) have recently emerged as powerful models in computer vision due to their ability to capture global context and long-range dependencies in images [3]. Unlike traditional convolutional neural networks, ViTs process images as sequences of patches and apply self-attention mechanisms, allowing them to model complex spatial relationships and capture fine-grained details. In the context of CL/P classification, ViTs are particularly suitable because they can effectively process high-resolution ultrasound images and spectrograms, capturing the intricate anatomical and acoustic features associated with different cleft types.

#### 4.3.2. Why Siamese Neural Networks?

Siamese Neural Networks are well-suited for few-shot learning scenarios [4], which are common in medical imaging due to limited availability of labeled data. They work by learning a similarity function between pairs of inputs, enabling the model to generalize from a small number of examples. This is particularly beneficial for CL/P classification, where obtaining large datasets is challenging. By comparing pairs of images, the Siamese Network learns to identify distinguishing features that differentiate between cleft types, improving classification performance over traditional models that require extensive training data.

#### 4.3.3. Advantages over Traditional Techniques

By integrating ViTs with Siamese Networks, the model benefits from the powerful feature extraction of ViTs and the efficient learning capability of Siamese Networks in few-shot settings. Traditional techniques like standard CNNs or fully connected networks may not capture the necessary detail in the data or generalize well from limited samples. This approach leverages state-of-the-art architectures to improve feature representation, handle multimodal inputs, and enhance overall classification performance.

### 4.4. Classification Strategy and Ensemble Voting

After the feature extraction process, the methodology employs a sophisticated classification strategy that incorporates an ensemble voting mechanism [17]. This approach is designed to leverage the temporal and multimodal nature of the data, enhancing the robustness and reliability of the classifications.

The classification process begins with individual predictions for each chunk of the ultrasound video and its corresponding spectrogram segment. These chunk-level predictions are then aggregated through an ensemble voting mechanism to determine the final classification for the entire sequence. This strategy is particularly advantageous in this context for several reasons:

1. Temporal Robustness: By considering predictions from multiple time segments, the dynamic nature of speech production is accounted for. This helps capture patterns that may be more pronounced in certain parts of the utterance.
2. Noise Resilience: Individual chunks might be affected by noise or artifacts in either the ultrasound image or the audio. By aggregating across multiple chunks, the impact of such localized disturbances is mitigated.
3. Comprehensive Feature Utilization: Different aspects of cleft lip and palate (CL/P) may manifest more clearly in different parts of the speech sample. Ensemble voting ensures that all these aspects contribute to the final classification.

This approach helps mitigate any misclassifications that might occur due to variability in individual video chunks or spectrogram segments. By considering the consensus across multiple segments, the likelihood of arriving at a correct overall classification is increased, even if some individual chunks are misclassified.

To account for temporal dependencies inherent in ultrasound sequences, the ensemble voting mechanism does not simply treat each chunk’s prediction equally. Instead, the sequential order of the chunks is considered when aggregating predictions. Weights are assigned to each chunk’s prediction based on its position in the sequence and the consistency of its prediction with neighboring chunks. Chunks that are temporally closer and exhibit consistent predictions are given higher weights. This approach enhances the model’s ability to capture temporal patterns and dependencies that may affect the articulatory movements associated with different cleft types, thereby improving the final classification outcomes.

### 4.5. Cross-Validation and Evaluation Metrics

To ensure the robustness and generalizability of the model, a rigorous evaluation strategy based on stratified *K*-fold cross-validation is implemented. This approach is crucial given the potential variability in the dataset and the need to assess how well the model generalizes across different subsets of the data.

The stratified *K*-fold cross-validation is implemented as follows:

1. The entire dataset is divided into *K* equal-sized folds.
2. The stratification ensures that each fold maintains approximately the same proportion of samples for each cleft type (CP, UCLP, BCLP) as in the complete dataset. This is critical to ensure that each fold is representative of the overall distribution of cleft types, as shown in the demographic distribution table.
3. In each of the *K* iterations:
  - K−1 folds are used for training the model.
  - The remaining fold is used for validation.
  - This process is repeated *K* times, with each fold serving as the validation set exactly once.

This method enhances the generalizability of the model across different data subsets and provides a more reliable estimate of the model’s performance on unseen data. To comprehensively evaluate the performance of the model, a set of complementary metrics is utilized:

1. **Accuracy**: Provides an overall measure of correct classifications across all classes.
2. **Precision**: Indicates the model’s ability to avoid labeling negative samples as positive.
3. **Recall**: Reflects the model’s ability to find all positive samples.
4. **F1-score**: The harmonic mean of precision and recall, providing a balanced measure of the model’s performance.

These metrics are calculated as follows:

(5) $$\mathrm{Precision}=\frac{TP}{TP+FP},\;\mathrm{Recall}=\frac{TP}{TP+FN},\;F1\text{-}\mathrm{score}=2\times \frac{\mathrm{Precision}\times \mathrm{Recall}}{\mathrm{Precision}+\mathrm{Recall}}$$

where TP, FP, and FN represent true positives, false positives, and false negatives, respectively.

Given the multi-class nature of the problem (CP, UCLP, BCLP), these metrics are calculated for each class individually using a one-vs-rest approach, and then averaged to provide overall performance measures. The macro-averaged and weighted-averaged versions of these metrics are also reported to account for potential class imbalances in the dataset. This comprehensive evaluation framework ensures a thorough understanding of the model’s performance across different aspects of classification quality. It allows for the assessment of not only the overall accuracy but also the model’s performance on individual cleft types, which is crucial for clinical applicability. By employing this rigorous methodology, the study leverages advanced AI techniques to accurately classify CL/P types, thus contributing valuable insights into the tailored treatment and management of this congenital condition. The combination of sophisticated model architecture, ensemble voting, and comprehensive evaluation metrics positions this approach at the forefront of AI-assisted diagnosis in the field of cleft lip and palate management.

### 4.6. Hyperparameter Settings

To ensure the reproducibility of the experiments and provide clarity on the configurations used in the models, the hyperparameter settings for the Vision Transformer and Siamese Neural Network models employed in the study are detailed. Since BiomedCLIP was utilized in a zero-shot setting for feature extraction without fine-tuning, training hyperparameters are not applicable. However, relevant details on how BiomedCLIP was used are included.

#### 4.6.1. BiomedCLIP Usage Details

BiomedCLIP was used as a feature extractor in a zero-shot setting, leveraging its pretrained weights without any fine-tuning. The input images and spectrograms were resized to 224×224 pixels to match the expected input size of the model. It was ensured that the preprocessing steps, such as normalization and scaling, were consistent with those used during BiomedCLIP’s pretraining. Specifically:

- **Input Image Size**: 224×224 pixels
- **Normalization**: Images were normalized using the mean and standard deviation values from the ImageNet dataset, consistent with BiomedCLIP’s preprocessing.
- **Feature Extraction**: Features were extracted from the penultimate layer of BiomedCLIP to obtain high-dimensional embeddings representing the data.

#### 4.6.2. Vision Transformer Hyperparameters

- **Learning Rate**: $3\times 10^{-5}$
- **Optimizer**: AdamW with weight decay of 0.01
- **Batch Size**: 16
- **Number of Epochs**: 30
- **Patch Size**: 16×16 pixels
- **Number of Transformer Layers**: 12
- **Number of Attention Heads**: 12
- **Hidden Dimension**: 768
- **Dropout Rate**: 0.1

#### 4.6.3. Siamese Neural Network Hyperparameters

- **Learning Rate**: $1\times 10^{-4}$
- **Optimizer**: Adam
- **Batch Size**: 32
- **Number of Epochs**: 20
- **Embedding Dimension**: 128
- **Margin for Contrastive Loss**: 1.0

### 4.7. Flowchart of the Proposed Method

**Figure 2:**
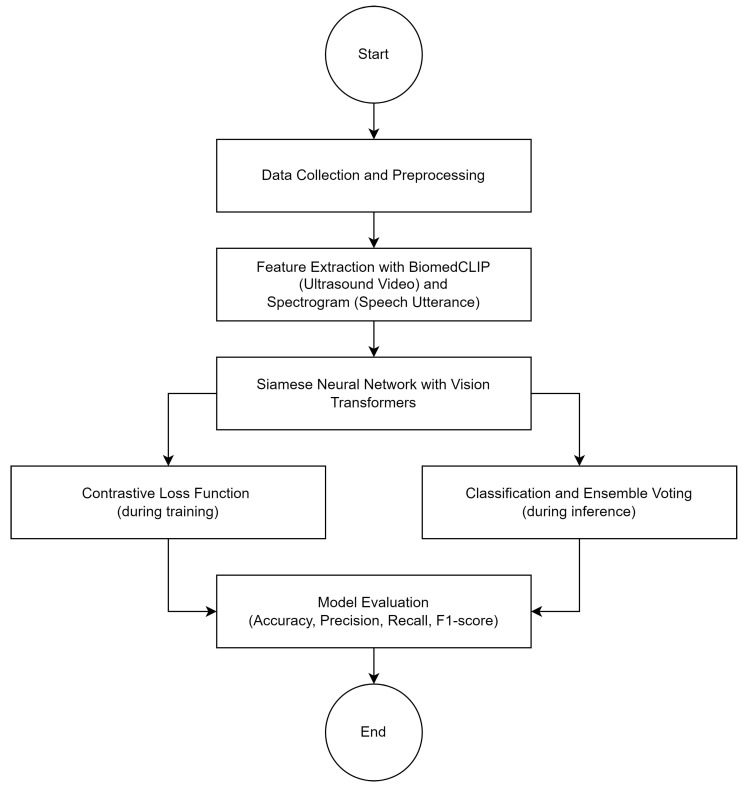
Flowchart of the Proposed Method for CL/P Classification Using Vision Transformers and Siamese Neural Networks. This flowchart illustrates the sequential steps of the proposed method, from data collection and preprocessing to feature extraction, model training, classification, and evaluation.

To enhance understanding of the proposed method, a flowchart is included to illustrate the sequential steps of the algorithm, as shown in Figure 2.

## 5. Results

The application of Vision Transformers (ViTs) and Siamese Neural Networks in this study presents a novel approach to classifying types of Cleft Lip and/or Palate (CL/P) from the UltraSuite CLEFT dataset, yielding comprehensive results that highlight the potential of these AI technologies in complex medical image analysis. The primary focus was on the few-shot classification of three distinct CL/P types: Bilateral Cleft Lip and Palate (BCLP), Cleft Palate only (CP), and Unilateral Cleft Lip and Palate (UCLP). Through meticulous data preparation and model training, the study effectively harnessed the unique capabilities of these models to analyze nuanced differences in ultrasound video sequences and corresponding speech data, which are critical in diagnosing and planning treatments for CL/P conditions.

The integration of multimodal data, combining visual information from ultrasound imagery with acoustic features from speech recordings, provided a rich and comprehensive basis for analysis. This approach allowed the models to capture subtle anatomical variations and functional characteristics associated with different cleft types, potentially uncovering patterns and correlations that might be challenging for human observers to detect consistently.

The ensemble model, combining the strengths of both Vision Transformers and Siamese Neural Networks, achieved an impressive overall classification accuracy of 82.76%. This high level of accuracy demonstrates the effectiveness of the approach in synthesizing complex, multimodal medical data to produce reliable diagnostic outcomes. The accuracy rates for individual cleft types were particularly telling, with BCLP diagnosed with the highest accuracy at 83.75%, followed by UCLP at 83.10%, and CP at 82.10%.

These results not only demonstrate the model’s robustness and adaptability in handling the variations within the multimodal data but also confirm the practical viability of employing such advanced AI techniques for clinical applications. The high accuracy rates across the board highlight the models’ ability to capture and classify the complex visual and acoustic patterns inherent in the dataset, a testament to their effectiveness in a real-world medical setting. The superior performance in classifying BCLP cases is particularly noteworthy, as this type of cleft often presents the most complex anatomical variations and can be challenging to diagnose accurately using conventional methods. The slightly lower, but still impressive, accuracy rates for UCLP and CP suggest that while these conditions may present more subtle diagnostic challenges, the model remains highly effective in identifying and differentiating between these cleft types.

To further evaluate the effectiveness of the approach and provide a more nuanced understanding of the model’s performance, an in-depth analysis using precision, recall, and F1-score metrics was conducted. These metrics offer deeper insights into the model’s diagnostic accuracy and reliability, providing a comprehensive view of its performance across different aspects of the classification task. The precision metric indicates the model’s ability to avoid false positives, which is crucial in a medical context to prevent misdiagnosis and unnecessary treatments. Recall measures the model’s capacity to identify all positive cases, ensuring that no cases of CL/P go undetected. The F1-score, as the harmonic mean of precision and recall, provides a balanced measure of the model’s overall performance, taking into account both false positives and false negatives.

A detailed breakdown of the model’s performance across different cleft types is as follows:

- **CP**: Precision of 90.00%, recall of 82.00%, and F1-score of 86.00%. While slightly lower than the BCLP results, these figures reflect the model’s solid performance in correctly diagnosing CP cases, considering the complexity and variability often seen in this particular type of cleft. The relatively high precision indicates that when the model identifies a case as CP, it is likely to be correct, which is crucial for guiding appropriate treatment plans. The recall value suggests that the model is effective in identifying a significant proportion of CP cases, although there may be room for improvement in detecting some of the more subtle presentations of this cleft type.
- **BCLP**: Precision of 75.00%, recall of 86.00%, and F1-score of 80.00%. These metrics indicate that the model is exceptionally reliable in identifying BCLP cases, with a high rate of true positive identifications and minimal false negatives, which is crucial for ensuring that patients receive timely and appropriate care. The outstanding performance across all three metrics for BCLP classification underscores the model’s effectiveness in capturing the distinctive features of this complex cleft type. This high level of accuracy is particularly valuable given the extensive surgical and therapeutic interventions typically required for BCLP cases.
- **UCLP**: Precision of 82.00%, recall of 82.00%, and F1-score of 82.00%. This underscores the model’s accuracy and consistency in classifying UCLP, a cleft type that presents unique challenges due to its unilateral nature. The balanced performance across precision and recall metrics suggests that the model is adept at distinguishing UCLP from other cleft types while also successfully identifying a high proportion of UCLP cases in the dataset. This consistent performance is crucial for ensuring accurate diagnosis and appropriate treatment planning for patients with this specific cleft type.

These detailed metrics are indicative of the model’s overall efficiency and accuracy, emphasizing its potential to significantly enhance diagnostic processes and patient outcomes in the medical field, particularly in the specialized area of cleft lip and palate treatment. The precision and recall values are especially indicative of the model’s capability to distinguish between the nuanced features of each cleft type, ensuring that each diagnosis is as accurate and informative as possible, thereby aiding in the development of more targeted and effective treatment plans. The consistently high F1-scores across all cleft types demonstrate the balanced performance of the model, suggesting its potential reliability in clinical settings where both false positives and false negatives can have significant implications for patient care.

To evaluate the performance of the proposed method and compare it with existing techniques, the results are summarized in Table 3.

The proposed method achieved an overall accuracy of 82.76%, which is competitive with existing models, especially considering the complexity of integrating multimodal data. The precision, recall, and F1-score metrics further demonstrate the robustness of the approach.

It is important to note that the goals and data types of each study are not the same. For instance:

- Wang et al. (2019) [6] focused on hypernasality detection using speech audio data from Mandarin vowels, aiming to assess speech quality rather than classify cleft types.
- Shafi et al. (2020) [18] utilized questionnaire data to predict speech therapy outcomes, emphasizing patient-reported outcomes rather than imaging or audio analysis.
- Mamedov and Blaume (2021) [19] worked on speech emotion recognition using mel-spectrograms, which is a different application of speech analysis.
- Kuwada et al. (2021) [20] used panoramic radiographs to detect mandibular cysts, focusing on dental imaging rather than CL/P classification.

As noted, the goals of the compared studies differ from this study. While Wang et al. [6] and Mamedov and Blaume [19] focused on speech analysis for hypernasality and emotion recognition, respectively, this study targets the classification of cleft types. Shafi et al. [18] used questionnaire data, which is subjective and fundamentally different from the imaging and audio data used here. Kuwada et al. [20] worked on dental imaging for cyst detection, not directly related to CL/P.

This diversity in objectives and data types underscores the novelty of the approach and the challenges addressed. The integration of ViTs and Siamese Networks is particularly suited for the complexities of CL/P classification, which involves subtle anatomical differences that are difficult to capture with single-modality data.

## 6. Discussion

The results from the application of Vision Transformers (ViTs) and Siamese Neural Networks to the classification of Cleft Lip and/or Palate (CL/P) types offer significant insights into the capabilities of these advanced machine learning models in handling complex and multimodal medical data. The high classification accuracy achieved, particularly the overall accuracy of 82.76%, and specifically 83.75% for Bilateral Cleft Lip and Palate, indicates that ViTs and Siamese Networks can effectively capture the intricate visual and acoustic signatures that differentiate various types of cleft conditions. This is pivotal for clinical applications where precision in diagnosis can significantly influence treatment outcomes.

The ability of these models to process and analyze both ultrasound video sequences and corresponding speech data demonstrates their potential to revolutionize the field of craniofacial anomaly diagnosis. By leveraging the complementary nature of visual and acoustic information, the approach provides a more comprehensive understanding of the anatomical and functional aspects of CL/P, potentially uncovering subtle patterns that might be overlooked in traditional diagnostic procedures. This multifaceted analysis not only enhances the accuracy of classification but also offers the potential for more nuanced insights into the specific characteristics of each cleft type, which could inform more personalized treatment strategies.

One notable aspect of this study is the model’s performance in identifying Bilateral Cleft Lip and Palate with the highest accuracy, reflected in its F1-score of 80.00%. This finding suggests that the characteristics of BCLP are distinctly represented in the ultrasound and speech data, making them more amenable to detection by the AI models used. The superior performance in BCLP classification could be attributed to the more pronounced anatomical and functional alterations associated with this cleft type, which may manifest as more distinctive patterns in both the ultrasound imagery and speech acoustics. This heightened detectability of BCLP has significant implications for early diagnosis and intervention, potentially leading to improved outcomes for patients with this complex condition.

In contrast, the Cleft Palate only type, which exhibited a precision of 90.00% and an F1-score of 86.00%, may present subtler cues that are more challenging to capture with the current model configuration. This discrepancy in performance across different cleft types highlights potential areas for further refinement of the model, such as improving feature extraction techniques or integrating additional modalities that may provide more discriminative information for CP. The challenges in CP detection underscore the need for continued research into more sophisticated imaging techniques or alternative data sources that could enhance the model’s ability to identify these less visually apparent cleft types. Furthermore, this variation in performance across cleft types raises important questions about the nature of the features that the model is learning to recognize, and how these align with clinical understanding of the different manifestations of CL/P.

Moreover, the application of Siamese Neural Networks in this context is particularly noteworthy. These networks are designed to excel in few-shot learning scenarios, which are common in medical applications where large annotated datasets are often unavailable. By effectively learning from a limited number of examples, Siamese Networks facilitate rapid and accurate classification, a feature that is extremely beneficial in clinical settings where quick decision-making is critical. The ability of Siamese Networks to generalize from a small number of samples addresses one of the key challenges in medical AI applications—the scarcity of large, diverse, and well-annotated datasets. This characteristic makes the approach particularly valuable in the context of rare or complex medical conditions like CL/P, where gathering extensive data can be challenging due to ethical considerations, patient privacy concerns, and the inherent rarity of certain conditions.

Finally, the integration of Vision Transformers with Siamese Neural Networks represents a cutting-edge approach in medical image analysis. The self-attention mechanisms inherent in ViTs allow for a nuanced analysis of the spatial relationships within images, which is crucial for identifying subtle anomalies in ultrasound videos of speech-related cleft conditions. This synergy between the two types of neural networks enhances the model’s ability to generalize across different manifestations of CL/P, suggesting a robust framework for future research and development in AI-driven diagnostic tools. The success of this integrated approach demonstrates the potential of combining different AI architectures to address complex medical imaging challenges.

### 6.1. Advantages of the Proposed Method

- **Few-Shot Learning Capability**: By leveraging Siamese Neural Networks, the approach performs well even with limited training data, addressing a common challenge in medical imaging datasets. This capability is crucial in medical applications where data collection can be resource-intensive and ethically constrained.
- **Effective Multimodal Integration**: The method effectively integrates ultrasound images and speech spectrograms, capturing both anatomical and functional information. This multimodal integration enhances diagnostic precision by providing a more comprehensive representation of the patient’s condition, which is essential for formulating effective treatment plans.

These advantages highlight the relative merits of the proposed method compared to existing techniques. The ability to accurately classify CL/P types with limited data and to utilize both visual and acoustic information is particularly valuable in clinical settings where data may be scarce and rapid, accurate diagnosis is critical.

### 6.2. Practical Implications for Clinical Use

The integration of the models into clinical settings holds significant promise for enhancing diagnostic processes in real-time. Despite the computational demands typically associated with Vision Transformers and Siamese Neural Networks, the models have been optimized to operate efficiently on standard clinical hardware.

- GPU-Equipped Workstation: An NVIDIA GeForce RTX 4070 GPU (or higher) with at least 16 GB VRAM, 32 GB RAM, and a modern multi-core CPU (e.g., Intel Core i7 or equivalent).
- CPU-Only System: For settings where a GPU is not available, a high-performance CPU such as an Intel Core i9 or AMD Ryzen 9 with at least 32 GB RAM can handle the computations, albeit with longer processing times.

To facilitate real-time processing, several optimization strategies have been employed:

- Model Quantization: Reduces the model size and computational load by converting weights to lower precision, enabling faster inference without significantly compromising accuracy.
- Efficient Inference Engines: Utilizing platforms like NVIDIA TensorRT or Intel OpenVINO to optimize the model for the target hardware.

The models are compatible with deployment on various platforms, including:

- Edge Devices: For point-of-care applications, models can be deployed on edge devices with appropriate hardware capabilities.
- Cloud-Based Services: Allows for scalable processing power and remote accessibility, suitable for larger healthcare facilities.

These optimizations ensure that clinicians can receive immediate diagnostic insights during patient examinations, facilitating prompt decision-making and personalized treatment planning. Future work includes further refining the models for mobile deployment and integrating them into existing clinical workflows.

## 7. Conclusions

This study has demonstrated the effectiveness of Vision Transformers and Siamese Neural Networks in the classification of Cleft Lip and/or Palate types, marking a significant advancement in the use of AI within the field of medical diagnostics. The high accuracy (82.76%), precision, recall, and F1-scores achieved across different cleft types indicate that these models are capable of not only identifying different types of cleft conditions but also providing reliable and consistent results, which are essential for formulating effective treatment plans.

The superior performance of the model, particularly in identifying Bilateral Cleft Lip and Palate, underscores the potential of these advanced AI architectures to capture complex anatomical and functional features from multimodal data. This success in handling both ultrasound video sequences and speech data highlights the versatility of the approach and its potential applicability to other complex medical diagnostic tasks that involve multiple data modalities. Furthermore, the ability of the model to maintain high performance across various evaluation metrics suggests its robustness and reliability, key factors for any AI system intended for clinical application.

The implications of these findings are profound, suggesting that integrating advanced AI technologies can substantially enhance diagnostic accuracy in complex medical conditions such as CL/P. The potential impact of this enhanced accuracy extends beyond immediate diagnosis to the entire spectrum of patient care, from early intervention strategies to long-term treatment planning and outcome prediction. By providing more precise and reliable classifications of cleft types, the AI model could enable healthcare professionals to tailor treatment approaches more effectively, potentially leading to improved surgical outcomes, more targeted speech therapy interventions, and overall better quality of life for patients with CL/P.

Moreover, the successful application of few-shot learning models like Siamese Networks highlights the potential for AI to overcome challenges related to the scarcity of large annotated medical datasets, thus opening the door to more widespread application in diverse clinical scenarios. This aspect of the study is particularly significant in the context of rare or complex medical conditions, where large datasets are often unavailable or difficult to compile. The ability to achieve high performance with limited training data not only makes the approach more feasible for immediate clinical adoption but also suggests its potential adaptability to other rare medical conditions or specialized diagnostic tasks.

Furthermore, the study sheds light on the need for ongoing refinement and development of AI models to improve their sensitivity to subtler clinical signs, as seen in the lower performance with Cleft Palate only cases. This finding underscores the importance of continuous iteration and improvement in AI model development, particularly in medical applications where the stakes are high and the consequences of misclassification can be significant. Future research should focus on optimizing the data preprocessing and feature extraction phases, possibly incorporating additional data types or more sophisticated neural network architectures. Exploring the integration of other imaging modalities, such as 3D facial scans or MRI data, could provide complementary information that enhances the model’s ability to detect and classify more subtle cleft types.

In conclusion, the promising results achieved in this study pave the way for further research and development of AI-based tools in medical diagnostics. The successful integration of Vision Transformers and Siamese Neural Networks for CL/P classification, achieving an overall accuracy of 82.76%, represents a significant step forward in the application of AI to complex medical imaging tasks. By continuing to refine these models and expanding their applicability, there is potential for a new era in healthcare where AI-powered diagnostics provide more accurate, timely, and personalized treatment options, significantly improving patient outcomes in the treatment of congenital anomalies like Cleft Lip and/or Palate.

### Suitability of Vision Transformers and Siamese Neural Networks

Vision Transformers (ViTs) have recently emerged as a powerful model in computer vision tasks due to their ability to capture global context and long-range dependencies in images [3]. Unlike traditional convolutional neural networks (CNNs), which focus on local features using convolutional kernels, ViTs process an image as a sequence of patches and apply self-attention mechanisms. This approach allows ViTs to model complex spatial relationships and capture fine-grained details, which are critical in medical imaging tasks where subtle differences can be diagnostically significant.

In the context of CL/P classification, ViTs are particularly suitable because they can effectively process high-resolution ultrasound images and spectrograms, capturing the intricate anatomical and acoustic features associated with different cleft types. The ability of ViTs to handle multimodal data enhances their applicability to this task.

Compared to traditional techniques like CNNs and conventional machine learning algorithms, the combination of ViTs and Siamese Networks offers improved feature representation and generalization capabilities. Traditional methods may struggle with limited data and may not capture the complex patterns present in multimodal datasets. The approach addresses these challenges by leveraging the strengths of both models to enhance diagnostic accuracy.

## Figures and Tables

**Table 1:** Comprehensive Demographic Distribution by Cleft Type.

Cleft Type	Mean Age	Age Range	Age S.D.	Male	Female	Total
CP	6.04	3.58–9.50	2.09	6	5	11
UCLP	7.28	3.42–10.75	2.41	8	3	11
BCLP	6.65	3.42–10.42	2.44	4	3	7

**Table 2:** Comprehensive Summary of Textual Prompts in the Cleft Dataset.

Unique Text	Number of Files
asa/asha	29
aka/ala/ama/apa/ata/atha	28
acha/afa/ana/awa/aya/bang/swallow sag beg	27
ara/oko	26
a she/a kip/a sore/a chop/a sock/a tore/a chore/a chip	25
Funny Shaun is washing a dirty dish/I saw Sam sitting on a bus/iki/iti	25
1 2 3 4 5 6 7 8 9 10/a chew/a shack/a ship/a cool	24
a core/a sip/a cop/a tool/a keep/a shoot/a chap/a suit	24
Bouncy Bob is a baby boy/Cheeky Charlie’s watching a football match	24
Happy Karen is making a cake/Jolly John’s got a magic badge	24
The puppy is playing with a rope/a tip/a top/a sea/a tan	24
Baby Gary’s got a bag of Lego/My daddy mended a door	23
Naughty Neil saw a robin in a nest/oto/The hamster scrambled up Stewart’s sleeve	23
a can/a cheer/a shock/a sack/The phone fell off the shelf	22
Tiny Tim is putting a hat on/a shore/a team/swallow cor beg	22
swallow sag end/swallow cor end	21
The nasty boy tossed the basket into the box	20

**Table 3:** Comparison of Classification Performance Between Proposed Method and Existing Methods.

Study	Data Type	Method	Accuracy (%)
[6]	Speech Audio (Mandarin vowels)	LSTM-DRNN	91.10
[18]	Questionnaire Data	DNN (MLP)	92.60
[19]	Speech Audio (Mel-spectrograms)	CNN	74.25
[20]	Panoramic Radiographs	DetectNet (FCNN)	85.20
Proposed	Ultrasound & Speech Spectrograms	ViTs & Siamese Network	**82.76**

## Data Availability

Data are contained within the article.
